# Autophagy biomarkers in CSF correlates with infarct size, clinical severity and neurological outcome in AIS patients

**DOI:** 10.1186/s12967-015-0726-3

**Published:** 2015-11-14

**Authors:** Honghong Li, Shuwei Qiu, Xiangpen Li, Mei Li, Ying Peng

**Affiliations:** Department of Neurology, Sun Yat-Sen Memorial Hospital, Sun Yat-Sen University, No. 107 West Yanjiang Road, Guangzhou, 510120 China; Guangdong Provincial Key Laboratory of Malignant Tumor Epigenetics and Gene Regulation, Sun Yat-Sen Memorial Hospital, Sun Yat-Sen University, Guangzhou, China; Department of Neurology, the First Affiliated Hospital of Soochow University, Suzhou, China

**Keywords:** Acute ischemic stroke, Autophagy, CSF, Outcome

## Abstract

**Background:**

Autophagy is demonstrated to be involved in acute ischemic stroke(AIS), which, however, is confined to cells and/or animals levels. The aim of this study was to determine two autophagy biomarkers, Beclin1 and LC3B, in cerebrospinal fluid (CSF) and serum of patients with AIS, and to evaluate a possible correlation between levels of Beclin1 and LC3B and severity of neurological deficit and clinical outcome of stroke patients.

**Methods:**

Levels of Beclin1 and LC3B were quantified by ELISA in CSF and serum collected from 37 AIS patients and 21 controls. The clinical severity at stroke onset was determined by the National Institute of Health Stroke Scale (NIHSS) and the neurological outcome was determined by the Modified Rankin Scale (mRs) and the improvement in NIHSS between stroke onset and 3 months later. Associations between autophagy biomarkers and infarct volume, NIHSS and mRs were assessed using Pearson analysis.

**Results:**

The levels of Beclin1 and LC3B were increased both in CSF and serum of AIS patients relative to controls. In CSF, they were positively correlated with infarct volume and NIHSS scores, and negatively correlated with mRs scores, but no significant association was observed in serum. Moreover, AIS patients with higher levels of Beclin1 and LC3B in CSF had significantly higher improvement in NIHSS.

**Conclusion:**

CSF and serum levels of autophagy biomarkers are altered in AIS patients. CSF levels of autophagy biomarkers are associated with infarct volume, clinical severity of and neurological outcome.

## Background

Acute ischemic stroke (AIS) is a major cause of death and disablement all over the world [1]. Despite its high prevalence and increasing burden, there are no effective neuroprotective agents in clinical use [2, 3]. The tissue plasminogen activator (tPA) is the only approved drug for AIS [4]. However, the intravenous thromnolysis is limited due to the strict therapeutic window and high risk of hemorrhagic complications [5]. Thus, exploration of effective therapeutic strategy becomes a major challenge [6]. AIS can initiate a series of biochemical reactions that directly or indirectly injury cellular structures [7, 8]. Many substances are released into the cerebrospinal fluid (CSF) and blood during brain damage.

Recent studies have revealed that autophagy may play an important role in ischemic stroke [9, 10]. Many investigations indicate that stroke induces activation of autophagy [11]. Autophagy is a critical cellular process responsible for the degradation and recycling of cellular components via the lysosomal pathway [12, 13]. This process is important for healthy cells to efficiently remove and recycle cellular constituents and maintain metabolic homeostasis [14]. Despite its essential role in cellular physiology, alterations in the process also operate as a pathological mechanism in many diseases, including brain damage [15, 16]. LC3B is a ubiquitin-like protein encoded by the mammalian homologue of autophagy associated gene 8 (Atg8) and is a reliable marker of active autophagosomes due to its tight correlation with numbers of autophagosomes [17, 18]. The synthesis of LC3B is increased during the process of autophagy, making it a key readout of levels of autophagy in mammalian cells [19, 20]. Beclin1, the mammalian homolog of yeast Atg6 and mammalian Vps15 [21], is an another marker of autophagy for its essential role for autophagy activation [22–24]. Some reports show that autophagy causes energy depletion, DNA fragmentation and severe damage in intracellular components [25–27]. By contrast, some other studies report the protective role of autophagy in ischemic injury [28–31]. Moreover, in vivo evidence from patients involving autophagy is still lacking and thus the role of autophagy in patients with ischemic stroke need more investigation [32–34].

The present study was designed to investigate the role of autophagy in AIS patients, with Beclin1 and LC3B used as markers of autophagy. Our study showed that levels of Beclin1 and LC3B greatly increased both in CSF and serum of patients with AIS. More interestingly, we found that Beclin1 and LC3B in CSF were correlated with infarct volume, clinical severity and neurological outcome.

## Subjects and methods

### Patients

This study was performed in Sun Yat-Sen Memorial Hospital of Sun Yat-Sen University from Mar. 2013 through Dec. 2014. To accurately evaluate the effect of autophagy on acute ischemia injury, the patients who met the following criteria were included: (1) the first ever stroke, without history of cerebrovascular disease; (2) within 24 h after onset; (3) age ≥18 years; (4) evidence of previous infarct lesion by brain computed tomography (CT) or magnetic resonance (MR); (5) willing to sign a informed consent document. The exclusion criteria were as follows: (1) concomitant systemic disease such as cardiovascular disease; (2) concomitant malignant disease; (3) history of diabetes; (4) history of surgery or trauma recently; (5) with autoimmune disease or treated with hormone or immuneinhibitors; (6) with neurological degenerative disease, such as Alzheimer’s disease, Parkinson’s disease, motor neuron disease, and multiple system atrophy; (7) receiving thrombolysis treatment. Forty-two AIS patients were included in the experimental group, and 5 patients were excluded due to the loss to follow-up when we assessed the mRS and NIHSS scores 3 months later after the stroke.

Detailed neurological examination and brain magnetic resonance imaging (MRI) 3.0T with diffusion weighted images (DWI) were performed in all AIS patients. Acute cerebral infarction was defined as an area of high signal intensity on the DWI. The edge of each infarct lesion was draw by the manual approach and the total infarct volume of each patient was calculated automatically by software Volume Viewer 2 (GE, AW Suite 2.0, 6.5.1.z) as previously described [35, 36]. To evaluate the severity of neurological deficit after AIS, a neurological deficit grading system National Institute of Health Stroke Scale (NIHSS) [37] was performed on each AIS patient within 24 h of stroke onset. The evaluation was performed immediately after blood and CSF samples were obtained and 3 months later it was repeated. The clinical outcome of AIS was determined by the Modified Rankins Scale (mRS) assessed 3 months after stroke and the improvement in NIHSS (ΔNIHSS) as previously described [38]. The ΔNIHSS was defined as the improvement in NIHSS = NIHSS^24h^ − NIHSS^3 months^ [38]. The MRI studies, NIHSS and mRS were evaluated by two neurologists who were blinded to the study.

The control subjects were selected from 21 age- and gender-matched patients with no central nervous system (CNS) disease, including psychoneurosis (n = 15), benign paroxysmal positional vertigo (n = 3), trigeminal neuralgia (n = 1), hypokalemic periodic paralysis (n = 1) and progressive muscular dystrophy (n = 1).

This study was approved by the Ethical Committee of the Sun Yat-Sen Memorial Hospital, Sun Yat-Sen University and conducted in accordance with the principles of the Declaration of Helsinki. All the participants were informed about the study and agreed to participate by signing an informed consent form.

### CSF and serum sample collection

CSF and peripheral blood samples of patients with AIS were collected within 10–24 h of the onset of neurological symptoms. CSF samples were taken during lumbar puncture between the L3/4 or L4/5 intervertebral space using a 25-gauge needle and held immediately on ice. Peripheral blood samples were collected by venepuncture in heparinized tubes and stored on ice. The CSF samples were centrifuged at 800 rpm for 5 min at +4 °C, and the blood samples were centrifuged at 3000 rpm for 15 min at +4 °C. All samples were stored at −80 °C until they were analyzed.

### Analysis of CSF and serum

Concentrations of two autophagy biomarkers, Beclin1 and LC3B, in CSF and blood were measured by commercial enzyme-linked immuno sorbent assay (ELISA) kits following the manufacturer’s instructions (SunLong Biotech Co., LTD, China). Briefly, for Beclin1 and LC3B evaluation CSF and serum samples were diluted 5 times. The optical density (OD) was measured by Microplate reader (Teng instrument co., LTD, USA) at a wavelength of 450 nm. A standard curve linear regression equation was calculated according to standards’ concentrations. Then, the concentrations of Beclin1 and LC3B in CSF and serum were calculated by the regression equation.

### Statistical analysis

All statistical analyses were performed by SPSS Statistics, version 19.0 software (SPSS, Inc, Armonk, NY). All graphs were generated by GraphPad Prism version 5.00 (GraphPad Software, San Diego,CA USA). All data were expressed as mean ± standard deviation (SD). Differences in characteristics between the AIS patients and controls were evaluated using the Student’s *t*-test for continuous variables or Chi square test for dischotomous variables. The Mann–Whitney *U* Test was used to compare the autophagy biomarkers between the AIS patients and controls. Differences in good outcome group and poor outcome group assessed by the improvement in NIHSS were evualted by the Student’s *t*-test. Pearson’s correlation test was used to assess the linear dependence between autophagy biomarkers and infarct volume, NIHSS and mRS. *P* <0.05 were considered to be statistically significant.

## Results

### Demographics

Patients were enrolled between Mar. 2013 and Dec. 2014. From the 42 AIS patients who were primarily included, 5 patients were excluded due to the loss to follow-up. Thus the final analysis was conducted on 37 AIS patients and 21 control subjects. Their demographic clinical characteristics are presented in Table 1. There were no significant differences in age, gender, blood pressure, cholesterol, low-density lipoprotein cholesterol (LDL-C), high-density lipoprotein cholesterol (HDL-C), triglyceride, and serum glucose between patients with AIS and controls.

**Table 1:** Demographics in AIS patients and controls (mean ± SD)

Variables	AIS (n = 37)	Con (n = 21)	*p* value
Gender			
Male	17	10	0.559
Female	20	11	
Age	59.38 ± 14.88	51.90 ± 15.80	0.078
SBP (mmHg)	130.19 ± 21.77	126.76 ± 20.46	0.558
DBP (mmHg)	81.27 ± 11.55	79.76 ± 9.98	0.618
Chol (mmol/l)	5.12 ± 1.31	5.22 ± 1.19	0.777
LDL (mmol/l)	3.13 ± 0.86	3.23 ± 0.75	0.652
HDL (mmol/l)	1.10 ± 0.30	1.14 ± 0.30	0.682
TG (mmol/l)	1.88 ± 1.54	1.92 ± 1.48	0.92
GLU (mmol/l)	4.93 ± 0.98	4.88 ± 0.73	0.852

*AIS* acute ischemic stroke, *Con* control, *SBP* systolic pressure, *DBP* diastolic pressure, *Chol* cholesterol, *LDL-C* low-density lipoprotein cholesterol, *HDL-C* high-density lipoprotein cholesterol, *TG* triglyceride, *GLU* glucose

### Beclin1 and LC3B concentrations

**Fig. 1:**
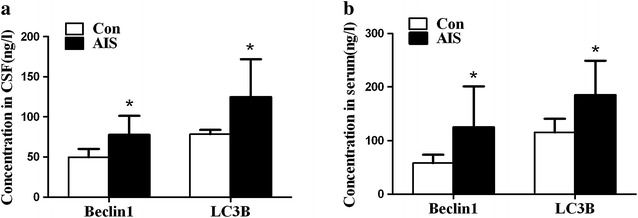
Levels of Beclin1 and LC3B in CSF (**a**) and serum (**b**) of controls (Con) and AIS. Concentrations of Beclin1 and LC3B both in CSF and serum were determined by ELISA (*p < 0.001)

In the observed population, samples were collected within 10–24 h of stroke onset. Firstly, we analyzed the change of levels of Beclin1 and LC3B both in CSF and serum of AIS patients. Concentrations of Beclin1 and LC3B in CSF and serum of AIS patients were significantly higher than that of control subjects (Fig. 1).

### Association between Beclin1 and LC3B and infarct volume and NIHSS

**Fig. 2:**
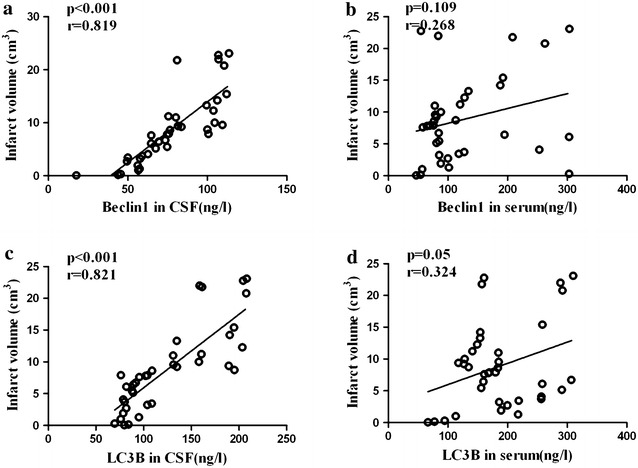
Associations between autophagy biomarkers and infarct volume (IV) in patients with AIS. The figures show **a** association between CSF Beclin1 and IV (*r* = 0.819, *p* < 0.001), **b** association between serum Beclin1 and IV (*r* = 0.268, *p* = 0.109), **c** association between CSF LC3B and IV (*r* = 0.821, *p* < 0.001), and **d** association between serum LC3B and IV (*r* = 0.324, *p* = 0.05)

**Fig. 3:**
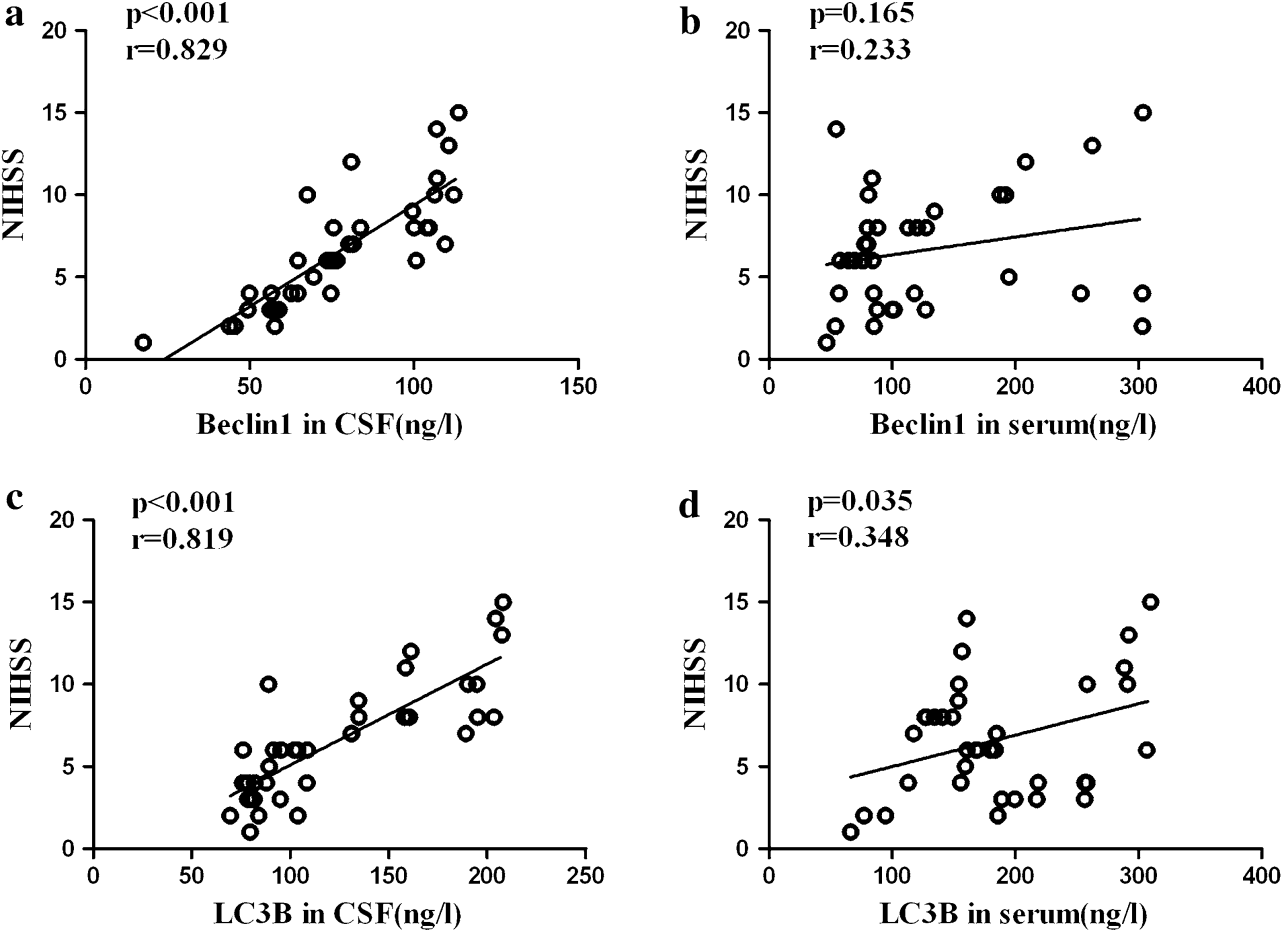
Associations between autophagy biomarkers and NIHSS scores in patients with AIS. **a**, **c** Correlation analysis between CSF Beclin1 and LC3B levels and NIHSS scores (Beclin1: *r* = 0.829, *p* < 0.001; LC3B: *r* = 0.819, *p* < 0.001). **b**, **d** Correlation analysis between serum Beclin1 and LC3B levels and NIHSS scores (Beclin1: *r* = 0.233, *p* = 0.165; LC3B: *r* = 0.348, *p* = 0.035)

We also investigated the association between autophagy and infarct volume and clinical severity of neurological deficit assessed by NIHSS in AIS patients. The mean infarct volume for all AIS patients was 8.82 ± 6.63 cm^3^. The mean NIHSS score for all AIS patients was 6.41 ± 3.578. In CSF, concentrations of Beclin1 and LC3B were positively correlated with infarct volume (Fig. 2a, c) and NIHSS (Fig. 3a, c). In serum, no significant association was observed between Beclin1 and LC3B levels and infarct volume (Fig. 2b, d), as well as the serum Beclin1 level and NIHSS (Fig. 3b). Although concentration of LC3B in serum was positively correlated with NIHSS (Fig. 3d), the correlation coefficient (r = 0.348) is low.

### Association between Beclin1 and LC3B and clinical outcome

**Fig. 4:**
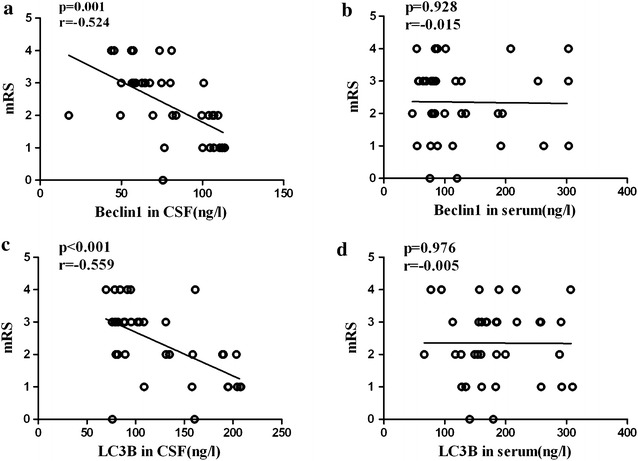
Associations between autophagy biomarkers and mRS scores in patients with AIS. **a**, **c** Correlation analysis between CSF Beclin1 and LC3B levels and mRS scores (Beclin1: *r* = −0.524, *p* = 0.001; LC3B: *r* = −0.559, *p* < 0.001). **b**, **d** Correlation analysis between serum Beclin1 and LC3B levels and mRS scores (Beclin1: *r* = −0.015, *p* = 0.928; LC3B: *r* = −0.005, *p* = 0.976)

**Fig. 5:**
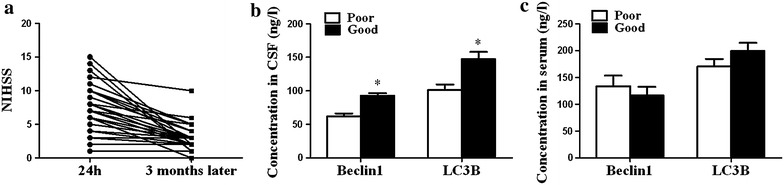
Relationship between autophagy biomarkers and outcome assessed by improvement in NIHSS in patients with AIS. **a** The NIHSS of AIS patients within 10–24 h of stroke onset and 3 months later. **b** CSF Beclin1 and LC3B levels in group of good outcome and poor outcome (Beclin1: *p* < 0.001; LC3B: *p* < 0.001). **c** Serum Beclin1 and LC3B in group of good outcome and poor outcome (Beclin1: *p* = 0.487; LC3B: *p* = 0.178)

Then, all the AIS cases were followed up for 3 months to investigate the role of autophagy in the outcome of AIS. The primary outcome was evaluated by mRS. In CSF, levels of Beclin1 and LC3B were negatively correlated with mRS (Fig. 4a, c), but no significant association was observed in serum (Fig. 4b, d). The improvement in NIHSS (ΔNIHSS = NIHSS^24 h^ − NIHSS^3 months^) was selected as the second outcome measures. ΔNIHSS ≥3 was considered to be good outcome and ΔNIHSS <3 was considered to be poor outcome. As a result, 19 cases of AIS patients gained good outcome and 18 cases gained poor outcome. We found that levels of Beclin1 and LC3B in CSF were significantly higher in good outcome patients than in poor outcome patients (Fig. 5a, c). However, there was no significant difference between levels of Beclin1 and LC3B in serum and improvement in NIHSS (Fig. 5b, d).

## Discussion

It was well demonstrated that autophagy participates in ischemic stroke [39–41], while the present study firstly reported the involvement of autophagy in patients with AIS. This study found that Beclin1 and LC3B were obviously increased in both CSF and serum of patients with AIS, and that levels of Beclin1 and LC3B in CSF were positively correlated with infarct volume and severity of neurological deficit. Moreover, levels of Beclin1 and LC3B in CSF were associated with good outcome of AIS patients.

In CNS, autophagy can be activated by cerebral ischemia/hypoxia, nutrient deprivation, oxidative stress, energy crisis and neurotoxins [42–44]. The initial role of autophagy activation is to provide proper cellular response for nutrients limitation [45, 46]. Autophagy is activated for lack of essential nutrients after ischemia [47]. Transition from basal level into induced autophagy could be regulated by multiple pathways in neurons during cerebral ischemic stroke [48]. Autophagy is activated not only by PI3 K-Akt-mTORC1 pathway [49], but also by AMP-activated protein kinase (AMPK) via activation of ULK1 or by inhibition of mammalian target of rapamycin complex (mTORC) [50]. Furthermore, increased AMP/ATP ratio and/or [Ca^2+^] activates AMPK kinase through activation of Ca2+/calmodulin-dependent protein kinase kinase (CaMKK) and LKB1 kinases, therefore inducing autophagy [51]. However, autophagy can be inhibited by the binding of Bcl-2 to Beclin1 via disrupting the association of Beclin1 with PI3K, hVps34 and p150 [52]. Consistent with observations, our study showed that autophagy was activated in serum and CSF of AIS patients with indicators increased, suggesting critical roles of autophagy in ischemic process.

In this study, levels of Beclin1 and LC3B in CSF were positively correlated with infarct volume and severity of neurological deficit, whereas this relation was not observed in serum. This is likely to be due to: First, the autophagy biomarkers in serum can not accurately reflect their levels in brain, and they may be affected by other organs of bodies when stroke takes place. Second, the sample size in this study was small.

More importantly, increased concentrations of Beclin1 and LC3B in CSF were found to be associated with good outcome, suggesting autophagy plays a protective role in AIS. Accumulating evidence suggests that autophagy enhanced functional recovery after stroke. It was repeatedly demonstrated that autophagy played a neuroprotective role in ischemic stroke. Urbanek et al. reported that rapamycin can effectively prevent neuro damage by induction of protective autophagy [53]. Autophagy inhibitor 3-MA can alleviate the neurological symptoms after ischemic stroke [16]. Also, Gao et al. demonstrated that ER stress-induced autophagy contributes to neuroprotective effect in cerebral ischemic preconditioning [54]. The protective role of autophagy in AIS was possibly attributed to elimination of damaged mitochondria and block of downstream apoptosis [41, 55]. However, it was also showed that autophagy could contribute to worse outcome [56, 57], and excessive activation of autophagy leads to neuronal death in ischemic stroke [58, 59]. In the neuronal system, moderate autophagy is thought to be neuroprotective [60], while excess or inadequate autophagy may promote neuronal cell death [61, 62]. In our study, the severity of neurologic deficit of most subjects was mild to moderate, so the moderate activation of autophagy exacted neuroprotective role in AIS patients.

There were some limitations in the present study. First, it is difficult to determine concentrations of Beclin1 and LC3B in CSF from normal persons as controls. Second, because of ethical concerns, our study was restricted to CSF and serum within 10–24 h of AIS, lacking of kinetic observation of alternations of Beclin1 and LC3B. Despite the limitations of the present study, our findings provide new evidence that autophagy was involved in AIS and was associated with clinical outcome.

In summary, we observed that autophagy biomarkers in CSF and serum levels of AIS patients were increased and that levels of Beclin1 and LC3B in the CSF were associated with good clinical outcome, implicating a thorough involvement of autophagy in ischemic injury and suggesting a bright intervention target in AIS treatment.

## Abbreviations

AIS: acute ischemic stroke
tPA: tissue plasminogen activator
LC3B: microtubule-associated protein B light chain 3
Atg: autophagy-related proteins
CT: computed tomography
MRI: magnetic resonance imaging
CNS: central nervous system
NIHSS: National Institute of Health Stroke Scale
mRS: modified Rankins score
CSF: cerebrospinal fluid
ELISA: enzyme-linked immuno sorbent assay
OD: optical density
SD: standard deviation
3MA: 3-methyladenine
SD: standard deviation
SBP: systolic pressure
DBP: diastolic pressure
TC: total cholesterol
HDL-C: high-density lipoprotein cholesterol
LDL-C: low-density lipoprotein cholesterol
mTORC: mammalian target of rapamycin complex
AMPK: AMP-activated protein kinase
CaMKK: Ca2+/calmodulin-dependent protein kinase kinase

## References

[1] Lakhan SE, Kirchgessner A, Hofer M (2009). Inflammatory mechanisms in ischemic stroke: therapeutic approaches. J Transl Med.

[2] Fisher M (2011). New approaches to neuroprotective drug development. Stroke.

[3] Moskowitz MA, Lo EH, Iadecola C (2010). The science of stroke: mechanisms in search of treatments. Neuron.

[4] Liang BA, Lew R, Zivin JA (2008). Review of tissue plasminogen activator, ischemic stroke, and potential legal issues. Arch Neurol.

[5] Yepes M, Roussel BD, Ali C, Vivien D (2009). Tissue-type plasminogen activator in the ischemic brain: more than a thrombolytic. Trends Neurosci.

[6] Almeida OP, Marsh K, Alfonso H, Flicker L, Davis TM, Hankey GJ (2010). B-vitamins reduce the long-term risk of depression after stroke: the VITATOPS-DEP trial. Ann Neurol.

[7] Fisher M, Schaebitz W (2000). An overview of acute stroke therapy: past, present, and future. Arch Intern Med.

[8] Neumar RW (2000). Molecular mechanisms of ischemic neuronal injury. Ann Emerg Med.

[9] Jiang T, Yu JT, Zhu XC, Zhang QQ, Tan MS, Cao L, Wang HF, Shi JQ, Gao L, Qin H (2015). Ischemic preconditioning provides neuroprotection by induction of AMP-activated protein kinase-dependent autophagy in a rat model of ischemic stroke. Mol Neurobiol.

[10] Pan R, Timmins GS, Liu W, Liu KJ (2015). Autophagy mediates astrocyte death during zinc-potentiated ischemia-reperfusion injury. Biol Trace Elem Res.

[11] Shang J, Deguchi K, Yamashita T, Ohta Y, Zhang H, Morimoto N, Liu N, Zhang X, Tian F, Matsuura T (2010). Antiapoptotic and antiautophagic effects of glial cell line-derived neurotrophic factor and hepatocyte growth factor after transient middle cerebral artery occlusion in rats. J Neurosci Res.

[12] Mizushima N, Levine B, Cuervo AM, Klionsky DJ (2008). Autophagy fights disease through cellular self-digestion. Nature.

[13] Kundu M, Thompson CB (2008). Autophagy: basic principles and relevance to disease. Annu Rev Pathol.

[14] Zhang T, Wang H, Li Q, Huang J, Sun X (2014). Modulating autophagy affects neuroamyloidogenesis in an in vitro ischemic stroke model. Neuroscience.

[15] Hu Z, Yang B, Mo X, Xiao H (2015). Mechanism and regulation of autophagy and its role in neuronal diseases. Mol Neurobiol.

[16] Yang Z, Zhong L, Zhong S, Xian R, Yuan B (2015). Hypoxia induces microglia autophagy and neural inflammation injury in focal cerebral ischemia model. Exp Mol Pathol.

[17] Kabeya Y, Mizushima N, Ueno T, Yamamoto A, Kirisako T, Noda T, Kominami E, Ohsumi Y, Yoshimori T (2000). LC3, a mammalian homologue of yeast Apg8p, is localized in autophagosome membranes after processing. EMBO J.

[18] Yorimitsu T, Klionsky DJ (2005). Autophagy: molecular machinery for self-eating. Cell Death Differ.

[19] Ichimura Y, Kirisako T, Takao T, Satomi Y, Shimonishi Y, Ishihara N, Mizushima N, Tanida I, Kominami E, Ohsumi M (2000). A ubiquitin-like system mediates protein lipidation. Nature.

[20] Barth S, Glick D, Macleod KF (2010). Autophagy: assays and artifacts. J Pathol.

[21] Hoyer-Hansen M, Jaattela M (2007). Connecting endoplasmic reticulum stress to autophagy by unfolded protein response and calcium. Cell Death Differ.

[22] Rami A, Langhagen A, Steiger S (2008). Focal cerebral ischemia induces upregulation of Beclin 1 and autophagy-like cell death. Neurobiol Dis.

[23] Diskin T, Tal-Or P, Erlich S, Mizrachy L, Alexandrovich A, Shohami E, Pinkas-Kramarski R (2005). Closed head injury induces upregulation of Beclin 1 at the cortical site of injury. J Neurotrauma.

[24] Fogel AI, Dlouhy BJ, Wang C, Ryu SW, Neutzner A, Hasson SA, Sideris DP, Abeliovich H, Youle RJ (2013). Role of membrane association and Atg14-dependent phosphorylation in beclin-1-mediated autophagy. Mol Cell Biol.

[25] Wen YD, Sheng R, Zhang LS, Han R, Zhang X, Zhang XD, Han F, Fukunaga K, Qin ZH (2008). Neuronal injury in rat model of permanent focal cerebral ischemia is associated with activation of autophagic and lysosomal pathways. Autophagy.

[26] Zhang T, Liu X, Li Q, Wang J, Jia W, Sun X (2010). Exacerbation of ischemia-induced amyloid-beta generation by diabetes is associated with autophagy activation in mice brain. Neurosci Lett.

[27] Puyal J, Vaslin A, Mottier V, Clarke PG (2009). Postischemic treatment of neonatal cerebral ischemia should target autophagy. Ann Neurol.

[28] Carloni S, Buonocore G, Balduini W (2008). Protective role of autophagy in neonatal hypoxia-ischemia induced brain injury. Neurobiol Dis.

[29] Wang P, Guan YF, Du H, Zhai QW, Su DF, Miao CY (2012). Induction of autophagy contributes to the neuroprotection of nicotinamide phosphoribosyltransferase in cerebral ischemia. Autophagy.

[30] Mehta SL, Lin Y, Chen W, Yu F, Cao L, He Q, Chan PH, Li PA (2011). Manganese superoxide dismutase deficiency exacerbates ischemic brain damage under hyperglycemic conditions by altering autophagy. Transl Stroke Res.

[31] Tanabe F, Yone K, Kawabata N, Sakakima H, Matsuda F, Ishidou Y, Maeda S, Abematsu M, Komiya S, Setoguchi T (2011). Accumulation of p62 in degenerated spinal cord under chronic mechanical compression: functional analysis of p62 and autophagy in hypoxic neuronal cells. Autophagy.

[32] Xu M, Zhang HL (2011). Death and survival of neuronal and astrocytic cells in ischemic brain injury: a role of autophagy. Acta Pharmacol Sin.

[33] Smith CM, Chen Y, Sullivan ML, Kochanek PM, Clark RS (2011). Autophagy in acute brain injury: feast, famine, or folly?. Neurobiol Dis.

[34] Rami A, Kogel D (2008). Apoptosis meets autophagy-like cell death in the ischemic penumbra: two sides of the same coin?. Autophagy.

[35] Tang Y, Rong X, Hu W, Li G, Yang X, Yang J, Xu P, Luo J (2014). Effect of edaravone on radiation-induced brain necrosis in patients with nasopharyngeal carcinoma after radiotherapy: a randomized controlled trial. J Neurooncol.

[36] Kim SH, Lee JY, Park SH, Jang HC, Lim EJ, Chang SJ, Lee SS (2013). Plasma B-type natriuretic peptide level in patients with acute cerebral infarction according to infarction subtype and infarction volume. Int J Med Sci.

[37] Tissue plasminogen activator for acute ischemic stroke (1995). The National Institute of Neurological Disorders and Stroke rt-PA Stroke Study Group. N Engl J Med.

[38] Yu SC, Kuo CL, Huang CS, Chang CS, Wu SL, Su SL, Liu CS (2012). Endogenous granulocyte colony-stimulating factor: a biomarker in acute ischemic stroke. Biomarkers.

[39] Wang P, Xu TY, Wei K, Guan YF, Wang X, Xu H, Su DF, Pei G, Miao CY (2014). ARRB1/beta-arrestin-1 mediates neuroprotection through coordination of BECN1-dependent autophagy in cerebral ischemia. Autophagy.

[40] Qi Z, Yan F, Shi W, Zhang C, Dong W, Zhao Y, Shen J, Ji X, Liu KJ, Luo Y (2014). AKT-related autophagy contributes to the neuroprotective efficacy of hydroxysafflor yellow A against ischemic stroke in rats. Transl Stroke Res.

[41] Zhang X, Yan H, Yuan Y, Gao J, Shen Z, Cheng Y, Shen Y, Wang RR, Wang X, Hu WW (2013). Cerebral ischemia-reperfusion-induced autophagy protects against neuronal injury by mitochondrial clearance. Autophagy.

[42] Levine B, Abrams J (2008). p53: the Janus of autophagy?. Nat Cell Biol.

[43] Mizushima N (2007). Autophagy: process and function. Genes Dev.

[44] Adhami F, Schloemer A, Kuan CY (2007). The roles of autophagy in cerebral ischemia. Autophagy.

[45] Yue Z, Friedman L, Komatsu M, Tanaka K (2009). The cellular pathways of neuronal autophagy and their implication in neurodegenerative diseases. Biochim Biophys Acta.

[46] Carloni S, Buonocore G, Balduini W (2008). Protective role of autophagy in neonatal hypoxia-ischemia induced brain injury. Neurobiol Dis.

[47] Mengesdorf T, Jensen PH, Mies G, Aufenberg C, Paschen W (2002). Down-regulation of parkin protein in transient focal cerebral ischemia: a link between stroke and degenerative disease?. Proc Natl Acad Sci USA.

[48] Gabryel B, Kost A, Kasprowska D (2012). Neuronal autophagy in cerebral ischemia—a potential target for neuroprotective strategies?. Pharmacol Rep.

[49] Carloni S, Girelli S, Scopa C, Buonocore G, Longini M, Balduini W (2010). Activation of autophagy and Akt/CREB signaling play an equivalent role in the neuroprotective effect of rapamycin in neonatal hypoxia-ischemia. Autophagy.

[50] Towler MC, Hardie DG (2007). AMP-activated protein kinase in metabolic control and insulin signaling. Circ Res.

[51] Hoyer-Hansen M, Bastholm L, Szyniarowski P, Campanella M, Szabadkai G, Farkas T, Bianchi K, Fehrenbacher N, Elling F, Rizzuto R (2007). Control of macroautophagy by calcium, calmodulin-dependent kinase kinase-beta, and Bcl-2. Mol Cell.

[52] Pattingre S, Tassa A, Qu X, Garuti R, Liang XH, Mizushima N, Packer M, Schneider MD, Levine B (2005). Bcl-2 antiapoptotic proteins inhibit Beclin 1-dependent autophagy. Cell.

[53] Urbanek T, Kuczmik W, Basta-Kaim A, Gabryel B (2014). Rapamycin induces of protective autophagy in vascular endothelial cells exposed to oxygen-glucose deprivation. Brain Res.

[54] Gao B, Zhang XY, Han R, Zhang TT, Chen C, Qin ZH, Sheng R (2013). The endoplasmic reticulum stress inhibitor salubrinal inhibits the activation of autophagy and neuroprotection induced by brain ischemic preconditioning. Acta Pharmacol Sin.

[55] Gabryel B, Kost A, Kasprowska D (2012). Neuronal autophagy in cerebral ischemia—a potential target for neuroprotective strategies?. Pharmacol Rep.

[56] Du L, Hickey RW, Bayir H, Watkins SC, Tyurin VA, Guo F, Kochanek PM, Jenkins LW, Ren J, Gibson G (2009). Starving neurons show sex difference in autophagy. J Biol Chem.

[57] Li J, McCullough LD (2010). Effects of AMP-activated protein kinase in cerebral ischemia. J Cereb Blood Flow Metab.

[58] Shi R, Weng J, Zhao L, Li XM, Gao TM, Kong J (2012). Excessive autophagy contributes to neuron death in cerebral ischemia. CNS Neurosci Ther.

[59] Ginet V, Spiehlmann A, Rummel C, Rudinskiy N, Grishchuk Y, Luthi-Carter R, Clarke PG, Truttmann AC, Puyal J (2014). Involvement of autophagy in hypoxic-excitotoxic neuronal death. Autophagy.

[60] Sheng R, Zhang LS, Han R, Liu XQ, Gao B, Qin ZH (2010). Autophagy activation is associated with neuroprotection in a rat model of focal cerebral ischemic preconditioning. Autophagy.

[61] Shacka JJ, Roth KA, Zhang J (2008). The autophagy-lysosomal degradation pathway: role in neurodegenerative disease and therapy. Front Biosci.

[62] Yue Z, Wang QJ, Komatsu M (2008). Neuronal autophagy: going the distance to the axon. Autophagy.

